# Hounsfield Unit on Preoperative Computed Tomography as an Indicator of Prognosis in Patients with Liposarcoma

**DOI:** 10.5152/tud.2024.24032

**Published:** 2024-05-01

**Authors:** Ryo Andy Ogasawara, Shugo Yajima, Naoki Imasato, Kohei Hirose, Ken Sekiya, Madoka Kataoka, Yasukazu Nakanishi, Hitoshi Masuda

**Affiliations:** 1 Department of Urology, National Cancer Center Hospital East, Chiba, Japan

**Keywords:** Computed tomography, dedifferentiated liposarcoma, prognosis, well-differentiated liposarcoma, x-ray

## Abstract

**Objective:**

Liposarcoma (LPS) is classified into 4 subtypes. As some subtypes have a high recurrence rate, knowing the risk of recurrence before surgery is important. Here, we aimed to investigate the relationship between Hounsfield units (HU) derived from preoperative computed tomography (CT) and the prognosis of patients undergoing surgery.

**Materials and Methods:**

We included 32 patients who underwent surgery for LPS between 2014 and 2022. Preoperative plain CT images were collected, and the HU value of each LPS was measured. The association between 2 HU categories (HU < cut-off vs. ≥ cut-off) and clinical variables was assessed. The optimal cut-off value was determined using statistical methods. We used the Kaplan–Meier method to determine the differences between the 2 HU categories at 2 endpoints: recurrence-free survival (RFS) and overall survival (OS).

**Results:**

The dedifferentiated subtype showed significantly higher HU values than the other subtypes (*P* < .001). The optimal cut-off value for HU was 20. HU < 20 was associated with young age, low-performance status, low Charlson Comorbidity Index, and well-differentiated pathology. The Kaplan–Meier curves demonstrated that RFS and OS were significantly shorter in patients with HU ≥ 20 than in those with HU < 20 (*P* = .007 and .04, respectively). However, when stratified based on subtype, no significant differences were observed between dedifferentiated and other subtypes.

**Conclusion:**

HU ≥ 20 on preoperative CT was associated with poor prognosis in LPS patients. Our findings suggest that preoperative CT-derived HU values may serve as useful predictors of prognosis.

**Main Points**

- Liposarcoma is a rare malignant tumor whose prognosis differs greatly between its 4 major subtypes: well-differentiated, dedifferentiated, myxoid, and pleomorphic.
- Dedifferentiated liposarcoma showed significantly higher Hounsfield unit (HU) on preoperative computed tomography than the other subtypes (*P* < .001).
- Recurrence-free survival and overall survival were significantly shorter in patients with HU ≥ 20 than in those with HU < 20 (*P* = .007 and .04, respectively).

## Introduction

Liposarcoma (LPS) is a rare malignant soft tissue tumor originating from fat cells. It accounts for approximately 15% of all soft tissue sarcoma subtypes, being the most common subtype among them.^1^ Histologically, LPS is classified into 4 major subtypes: well-differentiated LPS (WDLPS), also known as atypical lipomatous tumor, dedifferentiated LPS (DDLPS), myxoid LPS, and pleomorphic LPS.^2^ Well-differentiated LPS accounts for 40%-50% of all LPS. Compared with DDLPS, it is less likely to metastasize and has a relatively favorable prognosis. In contrast, DDLPS, accounting for 15%-20% of all LPS cases, is a high-grade and aggressive disease. DDLPS develops from WDLPS or sometimes arises de novo.^3^ Some DDLPS show a heterogeneous appearance, containing both well-differentiated areas and dedifferentiated lesions. It is associated with a high possibility of metastasis and local recurrence, which in turn leads to a poor prognosis. Recurrence-free survival (RFS) and overall survival (OS) at 5 years were significantly higher in WDLPS patients than in DDLPS patients (41.9% vs. 7.8%, *P <* .0001 and 92.1% vs. 36.5%, *P <* .0001, respectively).^4^

Surgical resection is the preferred treatment for LPS, if feasible, and the benefits of chemotherapy and radiation therapy remain uncertain.^5^ Computed tomography (CT) is one of the most common and efficient methods for diagnosing and evaluating LPS before surgery.^6^ The tumor’s Hounsfield unit (HU) value, measured using CT, varies with its composition.^7^ Fat has a low HU value; hence, LPS with a higher content of pure fat is likely to have a lower HU value.^8^ Therefore, we hypothesized that HU values on preoperative CT could indicate the level of LPS dedifferentiation or tumor aggressiveness. This retrospective study aimed to examine the relationship between tumor HU values and prognosis and histology in patients with LPS who underwent surgery.

## Materials and Methods

### Patient Selection and Study Design

We included 32 consecutive patients diagnosed with LPS who underwent surgery at our hospital between 2014 and 2022. All patients were included regardless of the primary tumor site (retroperitoneum, mediastinum, mesentery, or femur), but those with no preoperative CT images were excluded from the analysis. All 32 patients who underwent preoperative CT were included in the analysis. We used an opt-out approach in this retrospective study, and the institutional review board approved the study (2018-159). All the patients who participated in the study signed a written consent form for elective surgery.

### Radiological Data and Image Analysis

**Figure 1.**
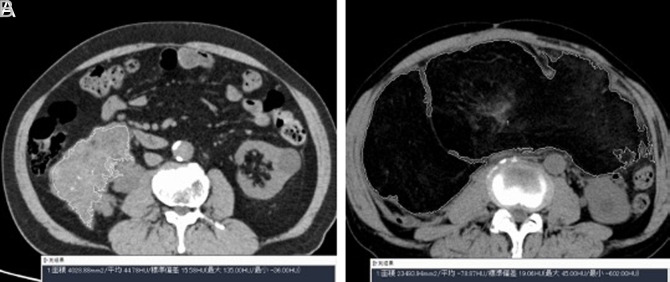
A and B. Representative examples of tumors and their corresponding CT images: A. Cases of dedifferentiated liposarcoma, with a higher HU value of 44.8. B. Case of well-differentiated liposarcoma, with a low HU value of −78.9. CT, computed tomography; HU, Hounsfield unit.

Preoperative abdominal CT images were automatically stored in the picture-archiving and communication system (PACS); images were evaluated using a PACS workstation (ShadeQuest/ViewR-DG; Fujifilm Medical Solutions, Tokyo, Japan). Preoperative abdominal non-contrast CT images were used to measure the HU. The boundaries of the tumors were automatically outlined using an automated segmentation program, and segmentation errors were corrected manually, if necessary. The PACS automatically measured HU as the mean value in the axial slice with the largest tumor diameter. Representative examples of CT images and HU measurements are shown in Figure 1. 

Urologists in their 4th and 11th year of practice, both of whom were blinded to patient information except for CT images, evaluated the images and measured HU to calculate inter-class correlation coefficients (ICCs).

### Data Collection

The following clinical data were collected and analyzed retrospectively: age, sex, body mass index (BMI), Eastern Cooperative Oncology Group performance status (ECOG-PS), Charlson comorbidity index (CCI), maximum transverse diameter of the tumor, pathological diagnosis (dedifferentiated, well-differentiated, myxoid, and pleomorphic), primary tumor site (retroperitoneum, mediastinum, mesentery, and femur), recurrence, and overall death. The pathological diagnosis was based on pathology reports written by experienced pathologists. 

### Endpoints

The primary endpoint was the association between the HU values and RFS. The secondary endpoint was the association between HU values and OS. Recurrence-free survival was defined as the period from the date of surgery to the detection of recurrence or the last follow-up date. Overall survival was defined as the period from the date of surgery to overall mortality, irrespective of the cause, or the last follow-up date. 

### Statistical Analyses

Continuous variables are presented as medians and interquartile ranges (IQR), and categorical variables are presented as numbers and percentages. The association between HU values and clinical variables was evaluated using the Mann–Whitney *U*-test for continuous variables and Pearson’s chi-square test or Fisher’s exact test for categorical variables.

Two-way ICC (2, 1) was calculated to evaluate the interobserver agreement of HU measurements between the 2 urologists.

The optimal cut-off value for HU was determined using receiver operating characteristic (ROC) analysis and the Youden index.

The Kaplan–Meier method and log-rank test were used to evaluate the differences in RFS and OS between the 2 HU categories (HU < cut-off vs. ≥ cut-off).

*P* values < .05 (two-sided) were considered statistically significant. We used the JMP 13 software (SAS Institute Inc., Cary, NC, USA) and GraphPad Prism 9.0 (GraphPad Software, Inc., San Diego, CA, USA) for statistical analysis and figure preparation, respectively.

## Results

### Patient Clinical Demographics

**Figure 2.**
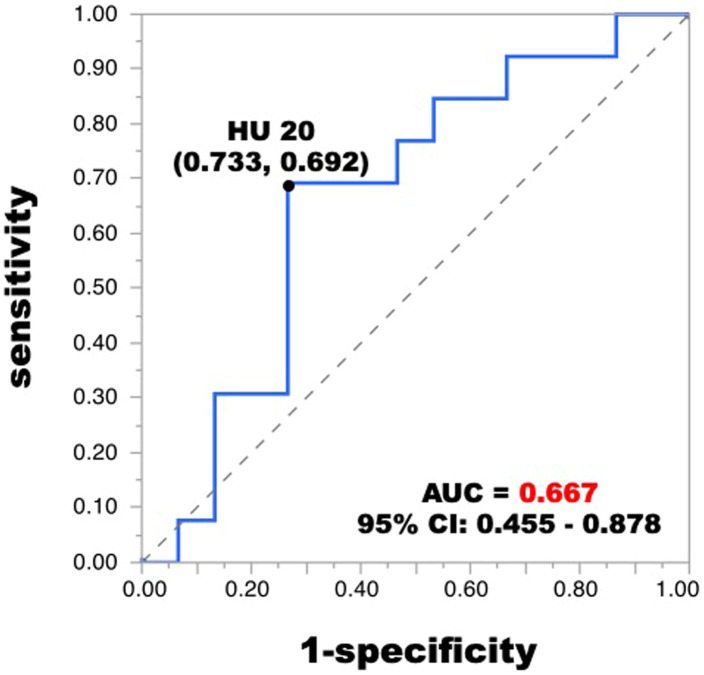
Receiver operating characteristic curve of HU for predicting postoperative recurrence. The optimal cut-off value for HU was 20 with the sensitivity of 0.692 and a specificity of 0.733 (AUC: 0.667, 95% CI (0.455-0.878)). ROC, receiver operating characteristic; HU, Hounsfield unit; AUC, area under the curve.

Hounsfield unit cut-off level was obtained by the ROC curve. The optimal cut-off value for HU was 20 with the sensitivity of 0.692 and a specificity of 0.733 (Figure 2, area under the curve: 0.667, 95% CI (0.455-0.878)).

Table 1 shows the clinical demographics of 32 patients stratified based on the 2 HU categories (HU < 20 vs. ≥ 20). Eighteen of the 32 patients had HU values less than 20, and 14 patients had HU values ≥ 20. The median patient age was 73 (IQR: 59-78) years. The group with a higher HU was significantly older than the group with a lower HU (69 [IQR: 53-76] vs. 77 [IQR: 70-79], *P* = .03). Nineteen patients (59%) were male. The median BMI was 20.4 (IQR: 18.5-22.4). While 4 of 18 (22%) patients in the lower HU group were graded as ECOG-PS 1, 9 of 14 (64%) patients in the higher HU group were graded as ECOG-PS 1, which was a significant difference (*P* = .03). The CCI was significantly higher in the higher HU group (0 [IQR: 0-0] vs. 1.5 [IQR: 0-2], *P* = .03). The median maximum transverse diameter of the tumor in the axial slice was 124 (IQR: 85-180) mm, which was not significantly different between the 2 groups (*P* = .20). Regarding the pathological findings, 16 of the 32 (50%) patients were diagnosed with DDLPS, and 11 (34%), 3 (9%), and 2 (6%) patients were diagnosed with well-differentiated, myxoid, and pleomorphic LPS, respectively. Of the 11 patients diagnosed with WDLPS, 10 (91%) had HU values of <20. No difference was observed in the primary tumor sites between the 2 groups. In total, 25 (78%) patients had retroperitoneal tumors, 1 (3%) had mediastinal tumors, 3 (9%) had mesenteric tumors, and 3 (9%) had femoral region tumors. Recurrence was observed in 15 (47%) patients during a median follow-up period of 761 (IQR: 406-2067) days. Of the 32 patients, 6 (19%) died during the follow-up period. 

### Treatment Details

No neoadjuvant therapy, such as chemotherapy or radiotherapy, was conducted preoperatively. Conventional open surgery was selected for 31 (97%) patients, and laparoscopic surgery was performed for only 1 (3%) patient. Adjacent organs were resected in 23 (72%) patients in which infiltration was detected preoperatively or direct invasion was observed during the operation. Microscopic analysis revealed a positive surgical margin in 9 (28%) patients and a negative margin in 7 (22%) patients. However, the surgical margin was not evaluated in 16 (50%) patients. As adjuvant therapy, 2 (6%) patients with residual tumors underwent radiotherapy. Salvage radiotherapy was performed in 4 (13%) patients with recurrent tumors. Although no patients underwent adjuvant chemotherapy, 3 (9%) patients were administered doxorubicin for recurrence.

### Relationship Between CT Findings and Pathology Results

**Figure 3.**
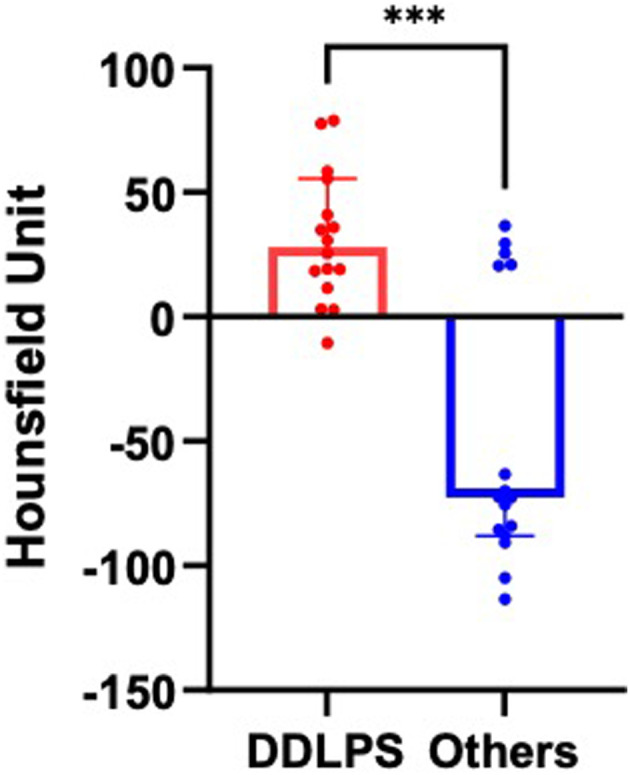
The differences in HU values on CT between dedifferentiated liposarcoma and other subtypes of liposarcoma are shown. The Mann–Whitney *U*-test revealed a significant difference between the 2 groups (*P* < .001). CT, computed tomography; DDLPS, dedifferentiated liposarcoma; HU, Hounsfield unit.

The HU value was significantly higher in DDLPS than in the other pathological subtypes of LPS (Figure 3, *P* < .001).

**Figure 4.**
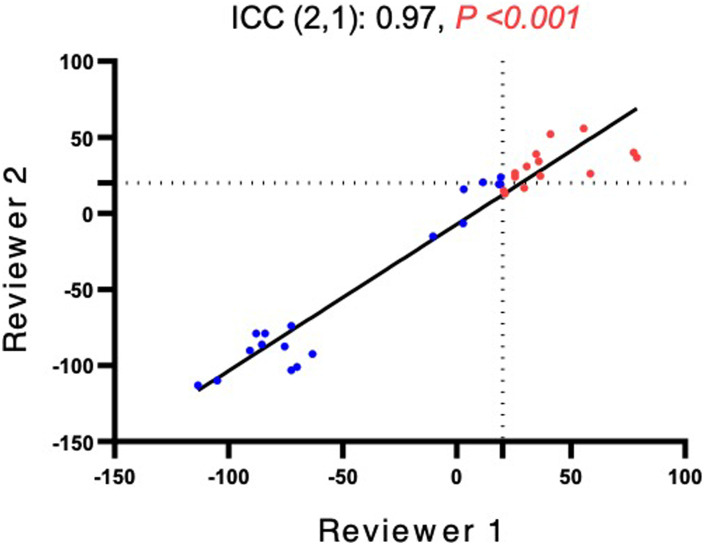
Interobserver variability of HU values between Reviewer 1 (urologist, 11 years of experience) and Reviewer 2 (urologist, 4 years of experience). The 2 reviewers had an excellent agreement, as indicated by ICC (2,1) = 0.97. The present validation used the measurement results obtained by Reviewer 1. HU, Hounsfield unit; ICC, intraclass correlation coefficients.

The interobserver ICCs for HU values were high at 0.97 (*P* < .001), which demonstrated high agreement between the 2 reviewers (Figure 4). 

### Survival

**Figure 5.**
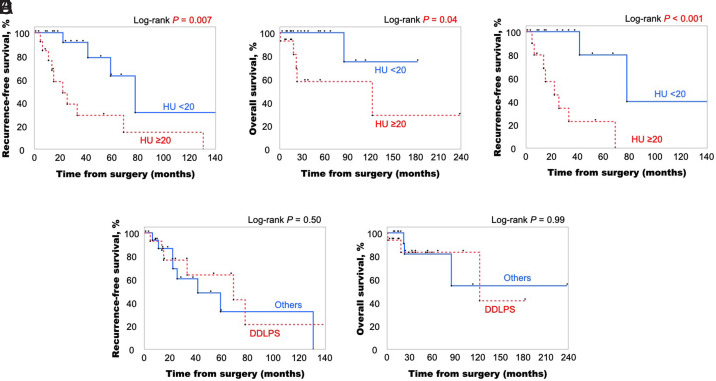
A-E. (A) Recurrence-free survival (RFS) and (B) overall survival (OS) Kaplan–Meier curves for a cohort of 32 liposarcoma (all types) patients stratified based on HU value cutoff of 20. The log-rank test showed that liposarcoma with HU values ≥20 had significantly shorter RFS (A) and OS (B) than those with HU values < 20. (C) Subgroup analysis of RFS within 25 retroperitoneal liposarcoma patients. The log-rank test showed that liposarcoma with HU values ≥20 had significantly shorter RFS than that with HU values <20. (D) Recurrence-free survival (RFS) and (E) overall survival (OS) Kaplan–Meier curves for a cohort of 32 liposarcoma (all types) patients stratified based on dedifferentiated and other pathologic subtypes. The log-rank test indicated no significant difference in RFS or OS between dedifferentiated and other liposarcoma subtypes. HU, Hounsfield unit; DDLPS, dedifferentiated liposarcoma.

Figure 5 shows Kaplan–Meier curves of RFS and OS stratified based on the 2 HU categories (HU < 20 vs. ≥ 20) for the whole cohort of 32 LPS patients. Patients with LPS whose HU values were ≥ 20 had significantly shorter RFS and OS than those with lower HU values (Figure 5A and B. log-rank test, *P* = .007 and .04, respectively). 

Subgroup analysis within 25 patients who had retroperitoneal LPS demonstrated significantly shorter RFS in patients with HU ≥ 20 than in those with HU < 20 (Figure 5C. log-rank test, *P* < .001).

However, when stratified based on DDLPS vs. other pathological subtypes, the Kaplan–Meier curves demonstrated no significant differences in RFS or OS (Figure 5D and E. log-rank test, *P* = .50 and .99, respectively).

## Discussion

Our findings suggest that LPS with an HU value ≥20 is a risk factor for future recurrence and mortality. Moreover, our findings demonstrated that the HU value was markedly elevated in DDLPS compared to that in the other pathological subtypes of LPS. This can be attributed to the percentage of fat components in the tumors. Well-differentiated LPS is usually characterized by abundant fat within the tumor, whereas DDLPS is characterized by a mixture of heterogeneous non-lipomatous and fat masses. Notably, 1 study evaluating the difference in CT images between WDLPS and DDLPS reported that DDLPS is significantly more likely to show focal/nodular water density, which is a nodular area with a high HU value similar to that of the muscle, than WDLPS.^9^ Myxoid and pleomorphic LPS contain less or no fat component.^6^ Given that WDLPS has a better prognosis than the others, a lower HU value, regardless of tumor subtypes, may reflect a higher percentage of pure fat and a lower percentage of dedifferentiated components within the tumor, which in turn leads to a better prognosis.

Several studies have reported factors that affect future recurrence and overall survival. Neuhaus reviewed the postoperative outcomes of 72 patients with retroperitoneal LPS and reported that low-grade histology and macroscopic complete resection were associated with a better outcome.^10^ In another study analyzing the outcomes of 177 patients, Singer also concluded that histologic subtypes and surgical margins were prognostic factors for survival.^11^ However, these factors were available only after surgery. Although histologic subtypes can be predicted through imaging and preoperative percutaneous biopsy, preoperative diagnosis is often inaccurate. One study analyzing 137 cases of preoperative percutaneous biopsy of retroperitoneal LPS reported an overall diagnostic accuracy of 62.8%, and the accuracy for identifying WDLPS was significantly higher than that for DDLPS (85.1% vs. 36.5%, *P* < .01).^12^

Unlike histological subtypes and surgical margins, HU values can be easily and accurately measured preoperatively. In this study, HU values were calculated automatically using PACS on the axial slice with the largest tumor diameter. Moreover, the interobserver ICC for the HU values was 0.97, thus indicating that the interobserver variability was small. 

Measuring the HU values before surgery can help clinicians make treatment plans and identify patients who require careful follow-up after surgery. As complete resection of the tumor is vital in LPS, the adjacent organs are often resected to achieve wide margins.^4^ However, if the HU values are <20 preoperatively, a less aggressive surgical approach may be possible. Even during postoperative follow-up, knowing the HU values may help in creating a flexible plan for follow-up, such as the frequency of imaging, based on the HU values of each patient. Patients with HU ≥ 20 may need closer follow-up due to the high probability of recurrence compared to those with HU < 20. 

Although our study revealed significant differences in RFS and OS when stratified based on HU values (HU < 20 vs. ≥ 20), no significant difference was observed when stratified based on pathological subtypes (DDLPS vs. others). This may have been due to the small cohort size. As the disease is rare and the study was designed as a single-center study, our cohort consisted of only 32 patients. Another possible explanation is that the “other pathological subtypes” contained not only WDLPS, which is known to show a relatively favorable prognosis, but also myxoid and pleomorphic LPS. Myxoid LPS has a better prognosis than DDLPS but not as favorable as WDLPS.^11^ Pleomorphic LPS is a high-grade tumor with a poorer prognosis than the other subtypes. It is characterized by its high risk of local recurrence and metastasis.^13^ Most of the “other subtypes” were WDLPS, but the inclusion of these two subtypes into the “other subtypes” category might have made it difficult to show a significant difference in RFS or OS when stratified based on pathological subtypes. 

Our study has some limitations. As this was a retrospective single-center study, our cohort may have been biased. The number of patients included was not as large as in previous studies, owing to the rarity of LPS.^10-12^ Moreover, no patients underwent any neoadjuvant therapy, and most of the patients did not undergo adjuvant therapy. Although the efficacy of chemotherapy and radiation therapy remains uncertain, they may affect patient prognosis.^14, 15^ Furthermore, although several studies have reported that surgical margin is the major prognostic factor, it was not fully evaluated in this study.^10, 11^ This may be because the peripheral tissue of surgical specimens was often so damaged that it was impossible for pathologists to assess the completeness of surgical resection. Finally, in our study, the high HU group exhibited high CCI and PS (Table 1). The heterogeneity of patient characteristics among the groups may have biased patient prognosis, especially OS. This study is exploratory in nature because of its small sample size, and a larger prospective trial is imperative to confirm our hypotheses.

Our study demonstrated that DDLPS had significantly higher HU values than the other pathological subtypes. Regardless of the LPS subtype, a higher HU value (≥20) was associated with a poorer prognosis. In conclusion, preoperative CT-derived HU values could serve as indicators of tumor aggressiveness and prognosis in patients with LPS and may provide clinicians with valuable insights for making treatment plans. However, future large cohort studies are essential to verify the findings of this study.

## Figures and Tables

**Table 1.** Clinical Demographics of 32 Patients Who were Diagnosed with Liposarcoma

Variables	Total (N = 32)	HU < 20 (N = 18)	HU ≥ 20 (N = 14)	*P*
Age, year	73 [59-78]	69 [53-76]	77 [70-79]	.03
Male	19 (59)	9 (50)	10 (71)	.29
Body mass index, kg/m^2^	20.4 [18.5-22.4]	20.2 [18.5-22.9]	21.0 [17.7-22.6]	.75
ECOG-PS				.03
0	19 (59)	14 (78)	5 (36)	
1	13 (41)	4 (22)	9 (64)	
Charlson Comorbidity Index	0 [0-2]	0 [0-0]	1.5 [0-2]	.03
Maximum transverse diameter of tumor, mm	124 [85-180]	144 [85-197]	105 [82-146]	.20
Pathological type				
Dedifferentiated	16 (50)	7 (39)	9 (64)	.15
Well-differentiated	11 (34)	10 (56)	1 (7)	.005
Myxoid	3 (9)	0	3 (21)	.07
Pleomorphic	2 (6)	1 (6)	1 (7)	1.00
Primary site				
Retroperitoneal	25 (78)	14 (78)	11 (79)	1.00
Mediastinal	1 (3)	0 (0)	1 (7)	.44
Mesentery	3 (9)	1 (6)	2 (14)	.57
Femoral region	3 (9)	3 (16)	0 (0)	.24
Recurrence	15 (47)	5 (28)	10 (71)	
Overall death	6 (19)	1 (6)	5 (36)	

Values in numbers (%) or median [interquartile range].

ECOG-PS, Eastern Cooperative Oncology Group performance status.
